# RETRACTED: Zhou et al. Pyrvinium Treatment Confers Hepatic Metabolic Benefits via β-Catenin Downregulation and AMPK Activation. *Pharmaceutics* 2021, *13*, 330

**DOI:** 10.3390/pharmaceutics15061623

**Published:** 2023-05-30

**Authors:** Shiwei Zhou, Obinna N. Obianom, Jiangsheng Huang, Dong Guo, Hong Yang, Qing Li, Yan Shu

**Affiliations:** 1 Department of Clinical Pharmacology, Xiangya Hospital, Central South University, Changsha 410008, China; 2 Department of Pharmaceutical Sciences, School of Pharmacy, University of Maryland, Baltimore, MD 21201, USA; 3 Department of Thyroid Surgery, The Second Xiangya Hospital, Central South University, Changsha 410011, China

The authors and the journal retract the article, ‘Pyrvinium Treatment Confers Hepatic Metabolic Benefits via β-Catenin Downregulation and AMPK Activation’ [1].

Following publication, the authors contacted the journal’s Editorial Office regarding two sets of mistakenly duplicated figures: Figure 3E is a copy of Figure 3D, and the loading control of Figure 4D is a copy of Figure 5A’s loading control.

Adhering to the journal complaints procedure, an investigation was conducted. The authors were unable to locate the original gel image of Figure 4D’s loading control data. The authors, the Editorial Board, and the Editor-in-Chief have agreed that the findings are no longer reliable, and the article was therefore retracted. 

This retraction was approved by the Editor-in-Chief of the journal *Pharmaceutics*.

The authors agreed to this retraction.
